# Supplementary material for: Baseline Plasma Tumor DNA (ctDNA) Correlates with PSA Kinetics in Metastatic Castration-Resistant Prostate Cancer (mCRPC) Treated with Abiraterone or Enzalutamide
Source: Cancers (Basel). 2022 Apr 29;14(9):2219. doi: 10.3390/cancers14092219 (PMC9102454; doi:10.3390/cancers14092219)
Supplement: Supplementary file 1 [file cancers-14-02219-s001.zip › cancers-1696594-supplementary.pdf]

Supplementary materials

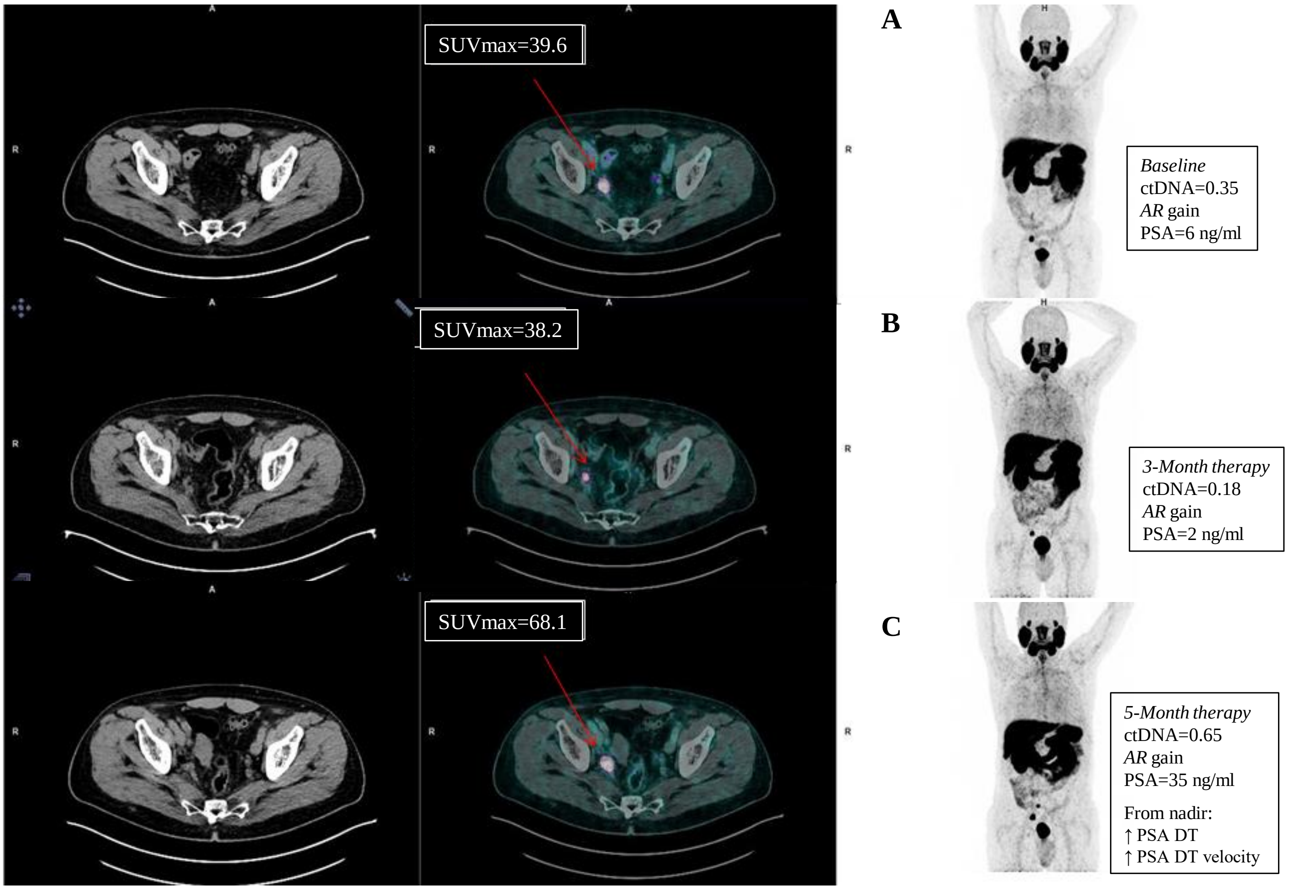

Figure S1. A representative case of the association between baseline high ctDNA and AR gain with increased PSA DT and PSA velocity at 5-month abiraterone treatment. Baseline ctDNA high and AR gain correlate with a shorter time to PSA, a faster PSA DT from nadir, as well as a significant higher PSA DT velocity from nadir. This patient belongs to the cohort characterized by a worst prognosis. *Abbreviations*: AR, androgen receptor; PSA DT, prostate specific antigen doubling time; ctDNA, plasma tumor DNA.

Table S1. Univariate analysis of PFS and OS

	PFS		OS	
	**HR (95% CI)**	**p**	**HR (95% CI)**	**p**
**logPSA** (continuous variable)	1.23 (1.14-1.32)	<0.0001	1.25 (1.16-1.35)	<0.0001
**Age** (≥74 vs <74)	1.03 (0.79-1.35)	0.803	0.98 (0.74-1.29)	0.881
**Bone metastasis** (yes vs no)	1.35 (0.91-2.01)	0.135	1.60 (1.05-2.45)	0.030
**Lymph nodes metastasis** (yes vs no)	1.01 (0.76-1.32)	0.978	0.92 (0.70-1.22)	0.586
**Visceral metastasis** (yes vs no)	1.87 (1.30-2.68)	0.0007	2.47 (1.71-3.58)	<0.0001
**Liver metastasis** (yes vs no)	2.59 (1.56-4.30)	0.0002	3.17 (1.89-5.32)	<0.0001
**Lung metastasis** (yes vs no)	1.24 (0.73-2.11)	0.416	1.73 (1.02-2.93)	0.043
**Other metastasis** (yes vs no)	1.66 (0.90-3.07)	0.104	1.96 (1.06-3.62)	0.032
**Previous therapy** (enza vs abi)	1.47 (1.11-1.94)	0.007	1.20 (0.90-1.59)	0.220
**Previous chemotherapy** (yes vs no)	1.98 (1.46-2.68)	<0.0001	2.11 (1.53-2.90)	<0.0001
**Prostatectomy** (yes vs no)	0.71 (0.54-0.94)	0.015	0.76 (0.57-1.01)	0.055
**Radioterapy** (yes vs no)	1.01 (0.74-1.38)	0.940	1.03 (0.74-1.42)	0.859
**ECOG PS** (2 vs 0-1)	3.26 (1.91-5.57)	<0.0001	5.88 (3.39-10.22)	<0.0001
**Gleason score** (≥8 vs 6-7)	1.10 (0.83-1.47)	0.513	1.05 (0.78-1.41)	0.765
***AR* CN** (Gain vs Normal)	2.66 (1.98-3.58)	<0.0001	3.02 (2.22-4.11)	<0.0001
**ctDNA** (>0.180 vs ≤0.180)	2.41 (1.71-3.38)	<0.0001	2.20 (1.55-3.12)	<0.0001
**ALP** (≥129 vs <129)	1.83 (1.39-2.42)	<0.0001	2.12 (1.59-2.83)	<0.0001
**Albumin** (<4 vs ≥4)	0.70 (0.53-0.92)	0.012	0.61 (0.46-0.81)	0.0007
**Hemoglobin** (<12.5 vs ≥12.5)	1.45 (1.08-1.94)	0.014	1.69 (1.24-2.30)	0.0009
**CgA** (≥120 vs <120)	1.49 (1.25-1.78)	<0.0001	1.83 (1.52-2.21)	<0.0001
**LDH** (≥225 vs <225)	2.61 (1.92-3.56)	<0.0001	2.68 (1.97-3.66)	<0.0001
**NLR** (≥3 vs <3)	1.50 (1.15-1.96)	0.003	1.47 (1.11-1.94)	0.006
**PSA DT from nadir** (continuous variable)	0.92 (0.89-0.96)	<0.0001	0.94 (0.91-0.98)	0.002
**PSA DT velocity from nadir** (continuous variable)	1.001 (1.001-1.002)	<0.0001	1.001 (1.001-1.002)	<0.0001

Abbreviations: ALP, alkaline phosphatase; AR CN, androgen receptor copy number; CgA, chromogranin A; ; ECOG, Eastern Cooperative Oncology Group; LDH, lactate dehydrogenase; NLR, neutrophil-lymphocyte ratio; PS, performance status; PSA DT, prostate specific antigen doubling time; ctDNA, circulating tumor DNA.
